# Influence of Core Starch and Lignocellulosic Fibers from Plantain (*Musa paradisiaca* L.) Pseudostem on the Development of Thermoplastic Starches and Biobased Composite Materials

**DOI:** 10.3390/polym17070859

**Published:** 2025-03-23

**Authors:** Andrés Mauricio Munar, Danilo Bonilla Trujillo, Nelly María Méndez, Carlos Guillermo Mesa, Paola Andrea Tenorio, Francisco Montealegre-Torres, Yean Carlos Zapata-Díaz, Lina Gisselth Ospina-Aguilar, Juan Pablo Castañeda-Niño

**Affiliations:** Escuela de Ciencias Agrícolas, Pecuarias y del Medio Ambiente, Universidad Nacional Abierta y a Distancia UNAD, Bogotá 111411, Colombia; danilo.bonilla@unad.edu.co (D.B.T.); nelly.mendez@unad.edu.co (N.M.M.); carlos.mesa@unad.edu.co (C.G.M.); paola.tenorio@unad.edu.co (P.A.T.); francisco.montealegre@unad.edu.co (F.M.-T.); yeanzapata@unicauca.edu.co (Y.C.Z.-D.); linaagui@unicauca.edu.co (L.G.O.-A.); juan.castaneda.nino@correounivalle.edu.co (J.P.C.-N.)

**Keywords:** plantain pseudostems, mechanical strength, steam explosion, *Musa paradisiaca* L.

## Abstract

As the demand for sustainable and environmentally friendly materials has increased, renewable resources have been explored for the development of biobased composites. Two biobased composite materials were developed from thermoplastic starch (TPS), short fibers from plantain pseudostems sheaths and the starch from the plantain pseudostem core, using twin-screw extrusion and compression molding. Based on the findings, there is evidence of a biobased composite material with reduced water absorption of up to 9.9%, keeping thermal stability at a degradation temperature between 300 and 306 °C and increasing tensile properties by over 506%, although hardness showed slight increases (4.6%). In addition, the capacity of the sheath to generate a water vapor barrier is highlighted by reducing the magnitude of losses in mechanical properties during storage for a period of 8 days. This study contributes to the use of agricultural residues to create sustainable products, offering a pathway toward reducing dependency on synthetic polymers and mitigating environmental impact.

## 1. Introduction

Stockpiling in landfills and bodies of water has increased due to petroleum-derived plastic containers used to store, transport, and preserve perishable products, thus polluting the environment on a daily basis [1]. Among the alternatives to counteract the negative effects on the environment are the generation of electricity from the combustion of plastic waste, the synthesis of oils through pyrolysis, and their substitution from the use of biodegradable and biobased materials [2,3,4]. Starch, proteins and lignocellulosic fibers mixed with plasticizers have been used as alternatives, finding the availability of glycerol, sorbitol, and isosorbide, among others [5,6,7] by applying methods such as compression/injection molding and extrusion to manufacture thermoplastic starches (TPSs) and biobased composite materials (BCMs) [8,9,10,11].

The aforementioned raw materials can be taken from plant tissues of diverse crops comprising cereals (corn and wheat), tubers (potato), roots (cassava), and fruits [12,13,14]. Some of the fruits include the plantain crop, whose pulp found in the bunch is mainly used for food consumption, while the other by-products from the mother plant are mainly used for animal feed and organic fertilizer production [15,16]. Furthermore, different macromolecules have been identified in each of the by-products, finding starch with thermal and enzymatic resistance characteristics (≈80% dry base) in the bunch pulp [17], starch (12.8 to 48.5% dry base), lignocellulosic fibers (≈42.4%), and pectin (≈10.0 to 14.6%) in bunch peels [15,18,19,20]. The previously mentioned macromolecules are found in the plantain pseudostem together with other macromolecules or substances in different concentration levels, depending on the tissue to be considered, the pseudostem outer layers, and the core. The sheath has a high water content with values above 90%, 2.2% of lignocellulosic fibers and 2.1% of sap and starch with values below 1%, in contrast to the core, which contains pectin, 6.4% of lignocellulosic fibers and 12.8% of starch [21,22,23].

To date, starches and fibers used to develop TPSs and BCMs have resulted in low mechanical properties, high water absorption, and low stability of mechanical, structural, and barrier properties during storage, which is considered undesirable for the development of new materials to manufacture food packaging and containers [13,24,25,26]. Nonetheless, an increase in mechanical and thermal properties through the manufacture of a bilayer material was identified, using a corn TPS layer and a plantain sheath layer, as an alternative to overcome the limitations of TPSs and BCMs [27]. The previous use of the pseudostem sheath to form a composite material can contribute to reinforcement through the long lignocellulosic fibers and serve as a coating for the TPS-based matrix, reducing the speed of water absorption and leading to a loss in mechanical properties. Its application, however, can be focused on the development of trays or semi-rigid packaging intended for the food sector.

Considering the physicochemical properties of the pulp, peel, and pseudostem sheath, a potential for their processing is identified to obtain a thermoplastic starch and a composite material based on plantain, considering the starch extraction from the pulp and peel, subjecting it to plasticization through a twin-screw extruder to obtain the TPS. To increase the mechanical properties and reduce the water absorption capacity, the plantain pseudostem sheaths were used with physical and thermal pretreatments for the elaboration of a biobased composite material using a sandwich-like structure by means of compression molding, generating a type of water vapor isolation by protecting the TPS from the storage environment. Finally, the mechanical, thermal, physicochemical, and structural characterization of the TPS and the biobased composite material was carried out considering a storage period of 8 days under controlled conditions. The purpose was to identify changes in the mechanical, thermal, physicochemical, and structural properties of water absorption in hygroscopic materials over a short-term period, as TPSs and BCMs were processed from raw materials with water contents below 6%.

## 2. Materials and Methods

Plantain bunches. Second-quality bunches of the Dominico varieties were harvested between the 17th and 18th week after flowering. Similarly, the pseudostem of the Dominico-Hartón variety was harvested after flowering in the same period. The bunches and pseudostems were collected in the municipality of Herveo, Department of Tolima (Colombia), organized by the Women’s Association of Herveo-La Esperanza (ASOMUHHES).

*Sodium metabisulfite* was supplied by Agenquímicos S.A.S. (Cali, Valle del Cauca, Colombia) at industrial grade.

Glycerin. USP-grade glycerol was used as a plasticizer as it is a polyol of functionality 3.0. This is a medium-viscosity, colorless liquid with 99.7% purity supplied by PROQUIM S.A.S (Cali, Colombia).

Starch extraction. Each finger of the rachis was first detached to be washed in neutral soap and water to remove farming and transport impurities. The plantain fingers were immersed in 1% ascorbic acid solution and then sliced with a knife to increase the surface area and reduce drying time by forced convection at a temperature of 70 °C over 24 h. When a moisture content of less than 8% was achieved in the resulting chips, the flour was processed using a knife mill with a 0.5 mm sieve. Once the flour from the plantain pulp and peel was ready, it was added to a 1.2% solution of sodium metabisulfite under constant agitation at 700 rpm for 1 h at room temperature. The resulting mixture was passed through a centrifuge to separate the slurry (starch and water) and bagasse (fibers, pectin, and parenchyma). The slurry resulting from filtration was stored in 12 L containers for 24 h to allow starch sedimentation and then decantated from the supernatant to obtain the wet starch paste. The starch paste was dried by forced convection using a temperature of 60 °C over 24 h, yielding starch agglomerate. To reduce and standardize the starch particle size, a knife mill with a 0.25 mm screen was used, followed by sieving with a 100 mesh/depth screen according to the Tyler series, resulting in particles with diameters of less than 150 µm [28].

Adjusting the plantain pseudostem sheaths. Pseudostems were collected on the same day of bunch harvesting, which began with the splitting of the sheath and the core. On the concave side of each sheath, the epidermis was removed and exposed to forced convection drying (Binder, FD 115, Germany) using a temperature of 60 °C over a period of 3 h to reduce the water content to 8% humidity [29] using a moisture balance (Radwag, MA 110.R, Poland).

**Figure 1:**
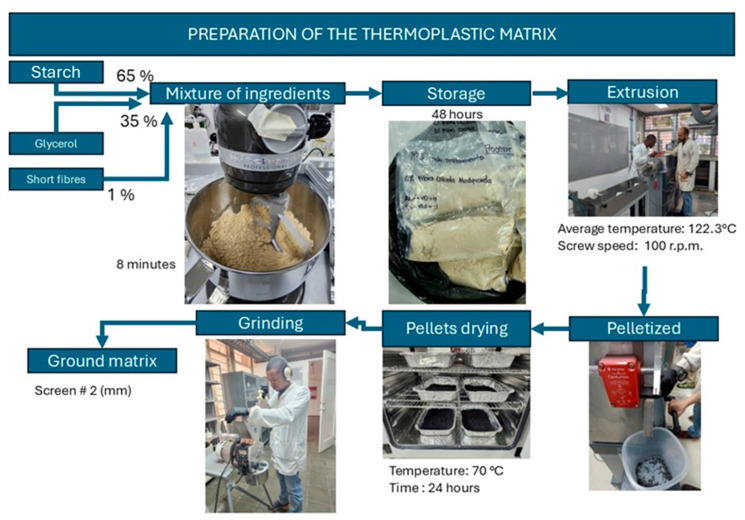
Processing of thermoplastic plantain starch.

Processing of thermoplastic starch (TPS). The native starch extracted from the plantain bunch (pulp and peel) had less than 6% water content and was mixed with glycerin at a mass ratio of 65/35, respectively. Mixing was performed with a KitchenAid Professional 600 mixer using the flat beater at level 2 speed for 8 min. The resulting blended paste was placed in high-barrier plastic bags and sealed to be stored for 48 h to enable the granules to be wetted with the plasticizer. The mixture was processed through a twin-screw extruder [Thermo Scientific, Haake Rheomex CTW 100 Polylab OS, Karlsruhe, Germany] to obtain plantain-based TPS pellets, with a mean temperature profile of 132 °C, a screw speed of 100 rpm, 2 mm nozzle extrusion die, and a 25/D ratio barrel for the extrusion of the bead. The TPS strand was pelletized, ground using a knife mill, and stored in high-barrier plastic bags [24,30]. Table 1 lists the TPS samples considered in this research. For TPS2, 1% of short fiber from the pseudostem sheath was added to the starch and glycerol mixture to be later extruded (Figure 1).

**Figure 2:**
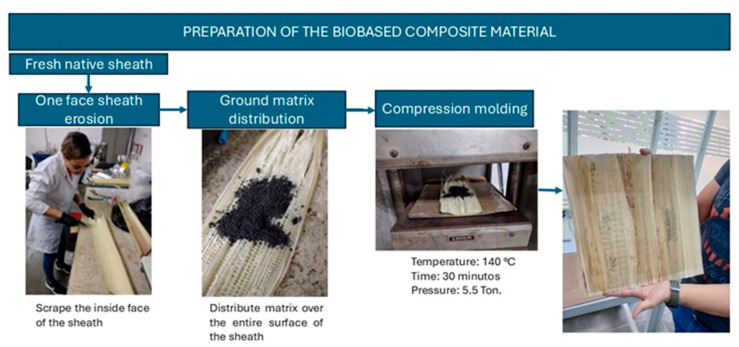
Production of biobased plantain composite material.

Preparation of biobased composite material. Having the TPS powder and treated sheaths, 20 g of the TPS (layer 2) was applied and distributed over the surface area of the treated sheath face (layer 1), followed by the laying of a second treated sheath (layer 3) on top of the TPS to create a sandwich-like structure of a biobased composite material. The layered structure consisting of TPSs and treated sheaths was placed in compression molding by applying a temperature of 140 °C and a pressure of 5.5 metric tons for a period of 15 min, followed by a cooling time of 10 min at the same pressure to obtain the biobased composite material with a sheet pattern structure (Figure 2). The above procedure is based on that reported by Rodríguez-Soto et al. (2019) [27] considering some variations, such as the use of a sheath layer to form the BCM.

### 2.1. Structural Characterization

Scanning electron microscopy (SEM). The samples were sized 1 cm × 3 cm × 5 cm. Each treatment was initially immersed in liquid nitrogen for 15 min and was then fractured by bending in order to expose and evaluate the cross-sectional area of each treatment. A cylindrical brass test tube was used with one end covered with graphite tape onto which the sample was placed. Using a Denton Vaccum Desk IV, USA, a gold coating was performed on the sample with a vacuum of 50 millitorr, and it was then placed in a sample holder and introduced into the chamber of a scanning electron microscope [JEOL model JSM 6490 LV, Tokyo, Japan]. A vacuum of 30 Pa was then applied to the SEM chamber, a common procedure for the use of backscattered electron radiation. When the vacuum stabilized, the tungsten filament was turned on, with an acceleration power of 15 kV [31]. Magnifications ranging from 200 to 1000× were used to take the micrographs.

### 2.2. Thermal Characterization

Thermogravimetric analysis. ASTM E 1131 was used for the thermal analysis of the sample measurements. An amount of 5 mg was collected from each sample, placed in a platinum capsule, and introduced into the muffle of the thermogravimetric analyzer (TA Instruments, Q50, New Castle, DE, USA) [32]. Each sample measurement was exposed to a temperature increase from 25 to 600 °C and a heating rate of 10 °C/min using a nitrogen atmosphere inside the chamber.

### 2.3. Physicochemical Characterization

Water absorption. From the TPS1, BCM1, and BCM2 treatments, the samples were cut into 2 × 2 cm specimens, dried at 80 °C for a period of 24 h, and then stored in a desiccator at 50% humidity using a solution of magnesium nitrate hexahydrate. Quantitative determination of water absorption was recorded using a microbalance during storage for 90 days [33]. A quintuplicate was used to assess water absorption in the four treatments. Statistical analysis involved determining normality using the Shapiro–Wilk test followed by ANOVA to evaluate significance (*p* < 0.05) between treatments. Levene’s test was applied to check for equal variances, and Dunnett’s T3 multiple comparison test was used for post hoc analysis using SPSS IBM software version 25.

### 2.4. Mechanical Characterization

Tensile test. Based on ASTM D638, the tensile test was performed with a universal testing machine (Tinius Olsen, model H50KS, Horsham, PA, USA) using a type IV test specimen. The distance between the jaws was adjusted to 58.25 mm, the speed of the moving jaw was adjusted to 5 mm/min, and the maximum force tolerance in the load cell was 500 N. The maximum tensile strength (σ_max_), modulus of elasticity (E), and strain at the breaking point (ε) were determined during storage at 50% relative humidity and a temperature of 23 ± 2 °C for 8 days [24,30]. A quintuplicate was used to determine water absorption in the four treatments.

Flexural test. Based on ASTM D790 and the use of a universal testing machine (Tinius Olsen, model H50KS, Horsham, PA, USA), the samples were rectangular in shape with a length of 75 mm, a width of 14 mm, and a thickness of 3 mm. Each sample or treatment was measured by placing it on two supports of the universal machine’s bending device at a distance of 4 cm and 1 cm from the ends, followed by the application of a bending force in the center of the test sample until reaching its fracture or a maximum deflection in bending of 5%. The test speed was 2 mm/min. Five specimens were evaluated per sample tested. The maximum flexural strength (σ_maxF_), maximum deflection in flexure (εF), and modulus of elasticity in flexure (EF) were determined for each sample during storage at a relative humidity of 50% and a temperature of 23 ± 2 °C for 8 days [34,35].

Hardness test. The procedure was performed using a Shore D durometer (Schmidt Control Instruments, Model HPSD-M, Waldkraiburg, Germany), and each recently processed sample was evaluated after being stored for 8 days at a relative humidity of 50% and a temperature of 23 ± 2 °C [36,37]. A quintuplicate was used to determine water absorption in the four treatments.

## 3. Results and Discussion

The TPSs and BCMs were subjected to a physicochemical, structural, thermal, and mechanical characterization, managing to differentiate the contribution generated by the adecuated sheaths and the starch from the plantain pseudostem core.

### 3.1. Structural Characterization

**Figure 3:**
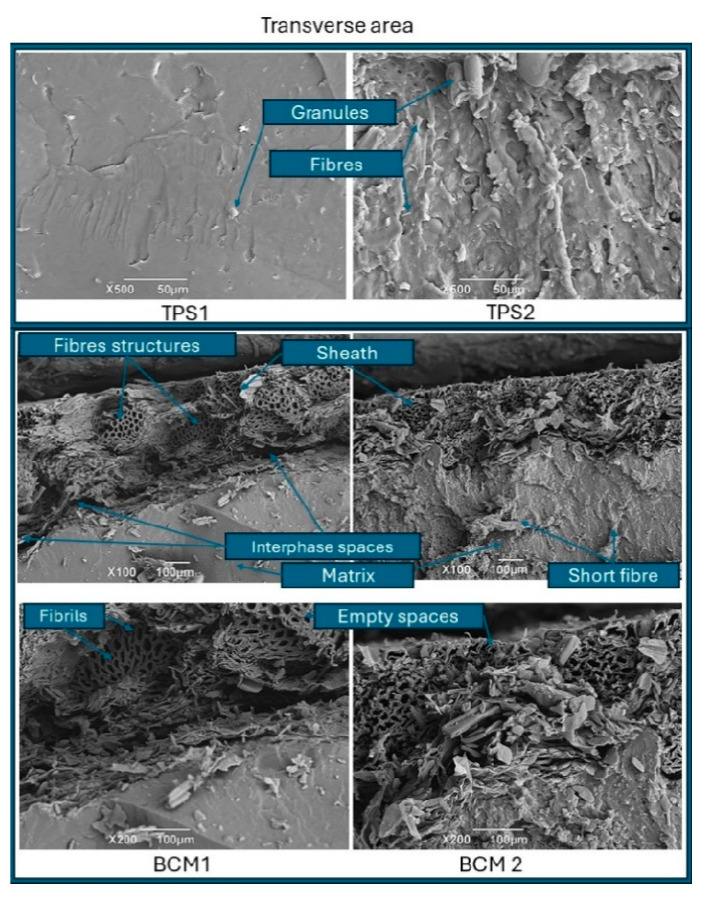
Micrographs of the TPS and BCM cross-sectional area.

Scanning electron microscopy (SEM). The cross-sectional area of the TPS showed two types of structures. TPS1 had a smooth surface with low porosity and a low concentration of native starch fragments, while TPS2 developed a higher degree of porosity when incorporating the starch and fiber from the pseudostem core (Figure 3).

In the case of Alanís-López et al. (2011) [38], when using native starch from Macho plantain for the preparation of a TPS by means of single-screw extrusion at an average temperature of 100 °C, a screw speed of 10 rpm, and using 40% glycerol and 10% water as plasticizers, greater roughness was observed due to the presence of larger portions of native granules with partial gelatinization, while Mendes et al. (2016) [39] mixed corn starch (60%), glycerol (24%), and water (15%) through a twin-screw extruder with an average temperature of 131.1 °C and a screw speed of 300 rpm, reaching a surface area similar to TPS1, thus generating a higher degree of breakdown and gelatinization of the starch granules.

When fibers are incorporated into the TPS matrix, Ma et al. (2005) [40] report that the fibers, in addition to reinforcing the matrix, affect the correct gelatinization of the starch granules as their concentration grows, causing an increase in their porosity (Figure 3).

**Figure 4:**
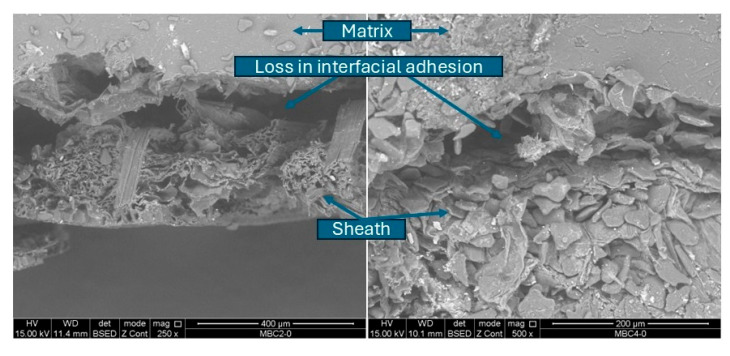
Biobased material composites aging on day 8.

Regarding composite materials, a superficial adherence between the matrix and the treated sheath was observed, showing the limited spacing between the two phases. The pseudostem fiber contains a trace of native starch without gelatinization and its structure shows cell walls, thus linking the structure of the lignocellulosic fibers with circular and/or cylindrical spacings, which is similar to the findings of Venegas et al. (2022) [41], who reported that the cross-section of plantain fibers are organized as a bundle of round and hollow fibrils joined together from the lignocellulosic complex. The above pattern can be related to the possible capacity to form hydrogen bridges between the plantain TPS-based matrix and the sheath since the polymeric chains of starch and cellulose can present secondary interactions through the hydroxyl groups found in both chemical structures [42,43]. When comparing the two composite materials, a greater number of empty spaces near the interfacial area was identified in BCM1, where the starch content of the core with its adhered fibers and pectin may contribute to a greater mechanical anchorage of the matrix with the sheath treated to be able to form BCM2. Holguín-Posso et al. (2024) [24] reported an increase in interfacial affinity between plantain peel fibers and the plantain TPS matrix by incorporating pectin through cocoa husk flour to form a biobased composite material from the processing sequence consisting of twin-screw extrusion, internal mixing, and compression molding. As day 8 of storage progressed (Figure 4), a detachment or weakening of the interface initially generated between the thermoplastic matrix and the plantain pseudostem sheath was identified due to the increase in water absorption.

### 3.2. Thermal Characterization

**Figure 5:**
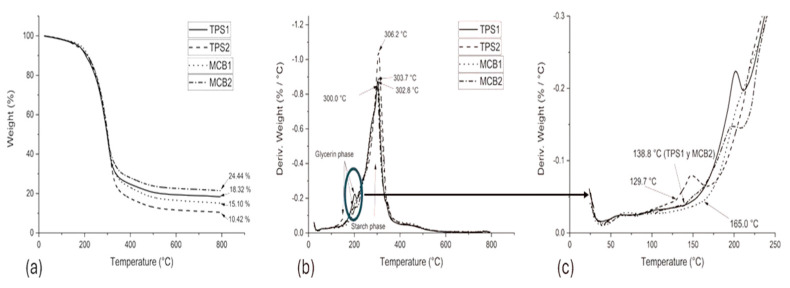
TGA thermograms in TPSs and BCMs. (**a**) TGA curves; (**b**) DTGA curves; (**c**) initial thermal degradation temperature.

Thermogravimetric analysis. DTGA thermograms made it possible to identify the phases with high glycerol content and those with high starch proportion in the TPS and BCM [33,44,45]. When both TPSs were transformed into BCMs, their glycerol phase was shifted to a higher temperature (Figure 5), which refers to an increase in thermal stability by incorporating short native fibers from the plantain pseudostem in the respective matrices and the forming of the sandwich structure using the pseudostem fibers, the former being involved in providing greater thermal protection to the higher glycerol content phase. Zhang et al. (2020) [46] reported that the phase with high glycerol or plasticizer content is mixed with water, indicating that the sheaths attached to the TPS reduce the rate of water absorption by the TPS.

Moreover, the initial temperature of thermal degradation (T_onset_) was identified (Figure 5), finding the above-mentioned pattern at 129.7 °C in TPS2, TPS1, and BCM2 were identified at 138.8 °C and BCM1 at 165.0 °C. According to this, despite evidencing the increase in each TPS and their respective BCM stability, a significant proportion of pectin from the core starch present in TPS2 may have increased the water absorption capacity towards the high glycerol phase, allowing an increase in the (T_onset_) temperature of BCM2 by 7.0% until achieving what was granted by TPS1, while the use of sheaths to coat TPS1 increased the thermal stability by 18.9%.

For the phase of the high starch content, reduced changes in temperature were observed, ranging from 300 to 306 °C, indicating that the two types of reinforcement used in the BCMs did not generate any type of thermal protection in the starch structures since Garcia-Ramon et al. (2021) [25] reported higher thermal stability from a thermal degradation at 370 °C in a BCM consisting of a TPS from plantain pulp starch reinforced with 4.1% cellulose nanoparticles from plantain rachis, while Venegas et al. (2022) [41] reported a value of 290 °C in a BCM made of plantain peel flour reinforced with 6.5% of short fibers from plantain pseudostem sheath.

Considering the aforementioned and as reported by Castañeda-Niño et al. (2024) [21], the plantain sheath fibers of the Dominico-Harton plantain variety contain 57.7% cellulose, 13.9% hemicellulose, and 27.7% lignin, which reveals that as the reinforcements are higher in cellulose content, the DTGA peak corresponding to the starch phase can be generated at higher temperatures [25,47]. Moreover, because of the empty spaces in the interior of the sheath structure, as evidenced by the SEM, no additional insulation or thermal resistance was observed. It is also important to note that the TPSs and BCMs were made from a mixture of pulp and core starches, resulting in a lower DTGA peak value than that reported for TPSs and BCMs processed from neat pulp starch.

### 3.3. Physicochemical Characterization

**Figure 6:**
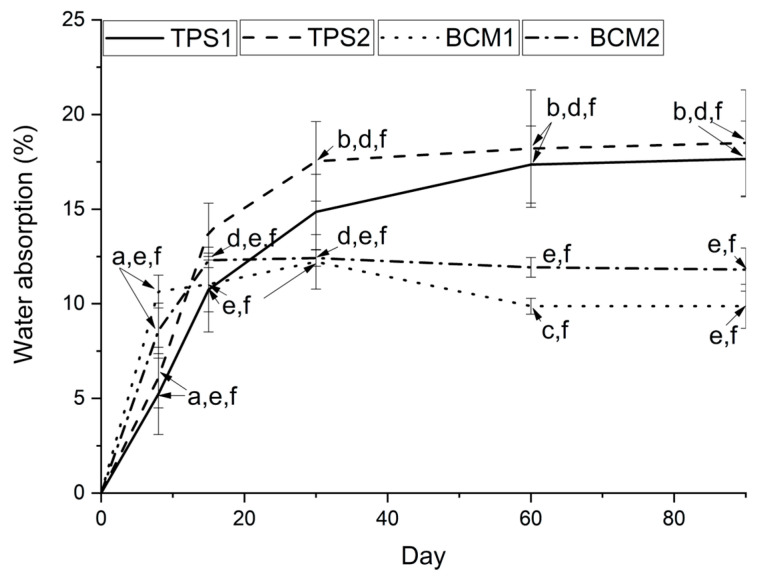
Water absorption in TPSs and BCMs. Significance identification for homogeneous subsets (**a**–**f**) among the four treatments over a 90-day storage period.

Water absorption. A high speed in the kinetics of water absorption was related to the first stage [33], between days 0 and 15, with the highest water absorption capacity in BCM1 followed by BCM2 and the lowest absorption speed in TPS1 and TPS2. This may be related to the greater water absorption capacity of the sheaths due to the presence of macromolecules of smaller size or molecular weight with the presence of hydroxyl groups, such as pectin, and the sap present in their structure when compared to TPS1 and TPS2 [21,48,49]. Nonetheless, the biobased composite materials reached a saturation point in water retention for the corresponding structures, with a maximum value of 12.2% for BCM1 and 12.4% for BCM2 on day 30, while the pair of TPSs had a continuous water absorption capacity of 14.9% in TPS1 and 17.5% in TPS2 (Figure 6). The above patterns are probably due to the higher availability of hydroxyl groups in the polymeric starch chains in TPS1 to further generate hydrogen bridges with the water available in the air during storage [50,51,52], differing from the composite materials, as their respective TPS matrices are protected by a possible action as a barrier to water vapor generated by the pseudostem sheath. In the case of TPS2, its water absorption kinetics were greater than those of TPS1, possibly due to the presence of remaining pectin in the core starch.

A second stage of water absorption is related to the reduction in water absorption kinetics, which occurs until the sample weight stabilizes after reaching maximum water retention in its structure [33]. The TPS reached stabilization by day 90, with a water absorption of 17.7%, whereas the composite materials did not exhibit the same stabilization pattern over a prolonged period. Instead, they showed a gradual reduction in water content, reaching values of 9.9% (BCM1) and 11.8% (BCM2) by day 90, indicating possible desorption. These water absorption values were lower than, or comparable to, those of composite materials made from epoxy resins and polyester matrices mixed with plantain pseudostem fibers at concentrations ranging from 16% to 40%, which yielded values between 21.0 and 29.9% and between 10.0 and 27.0%, respectively [41]. Despite the higher water absorption capacity of TPSs compared to BCMs due to the higher content of plasticized starch—especially in treatments with core starch, which had low purity because of the presence of pectin portions [21,53,54]—significant differences were observed through ANOVA and Dunnett’s T3 multiple comparison test. These differences were most pronounced when comparing water absorption during storage over two periods, from day 0 to day 15 and from day 60 onward, as the TPS demonstrated a greater capacity for absorbed water compared to the BCM (Figure 6).

### 3.4. Mechanical Characterization

**Figure 7:**
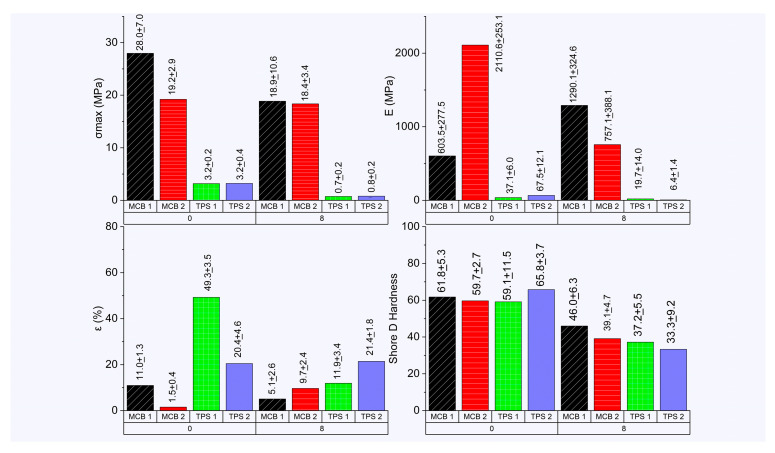
Tensile and hardness properties of TPSs and BCMs.

Tensile test. The TPS showed σ_max_ values ranging between 3.2 and 3.2 MPa, E between 37.1 to 67.5 MPa, and ε between 20.4 and 49.2% on the processing day (day 0), for which the highest values in σ_max_ and E, including the lowest value of ε, were found in TPS2, suggesting a possible reinforcement contribution by implementing 5% of the core starch (Figure 7) given the presence of lignocellulosic fibers and pectin entangled that adhered to the starch. Comparing the TPS and the plantain biobased composite material developed by Holguín-Posso et al. (2024) [24], higher values of σ_max_ were reported on day 0, showing 4.1 MPa in the TPS made of plantain pulp starch and 35% glycerin, while the composite material made of former TPSs and 30% plantain peel fibers reached 5.1 MPa, and in a second biobased composite material consisting of the TPS, 15% plantain peel fiber, including a portion of pectin and lignocellulosic fibers from 15% cocoa husk flour, increased this tensile property to 7.6 MPa. Similarly, E on day 0 was higher concerning this research, featuring values between 142.5 and 192.4 MPa, thus linking its greater tensile properties to the use of only pulp starch for the development of the matrix, as it has a high amylose content (28.4%) and has characteristics of resistant starch [55,56].

Regarding composite materials consisting of a sandwich-like structure between two treated sheaths and a TPS film, increases in σ_max_ were generated by 782.6% in BCM1 and 506.0% in BCM2 and E, with increases of 1526.2 and 5587.4%, respectively (Figure 7). These increases in the tensile properties are due to the mechanical contribution provided by the sheaths since a set of long fibers oriented in the direction of the force applied in the tension and the short fibers present in the respective matrices can be found for the increase in the tensile properties. Comparing the two composite materials and evidencing structural differences from the presence of a lower interfacial adhesion between the sheath and the matrix of BCM1, as shown by SEM, the highest E was generated by BCM2. It is important to mention that the values of the tensile properties provided by the two composite materials were comparable with some of the synthetic polymers (epoxy resins, polyester resins, high-density polyethylene, polypropylene, polystyrene, and nylon) reinforced with plantain pseudostem fibers with concentrations between 3.7 and 50%, reaching σ_max_ values between 5.2 and 26.0 MPa. E has been found between 0.15 and 1.8 GPa, while the reports of ε have been between 1.4 and 25% [57].

Furthermore, when compared with other biobased composite materials, it was observed that σ_max_ and E values were higher than those that were reinforced with short fibers, with lengths between 600 and 1200 µm, containing between 6 and 20% of lignocellulosic fibers or cellulose mixed with wheat, corn, or cassava starches and processed by extrusion, injection molding, or compression molding, generating σ_max_ values between 5.0 and 15.5 MPa [40,45,58,59]. Rodríguez-Soto et al. (2019) [27] reported developments related to the manufacture of BCMs considering the bilaminar structure from the use of a matrix consisting of 95% corn TPS and 5% polylactic acid (PLA), adhering to a plantain pseudostem sheath using compression molding and achieving σ_max_ from 50 to 52 MPa and E from 2.7 to 2.7 GPa. Concerning ε in the composite materials, a restriction of the macromolecular movement was generated from the interface generated between the treated sheath and the matrix since its value was reduced from 5.08 to 9.66%. This reduction in ε in the BCMs was also evident in other studies of the tensile test in plantain-based BCMs [24,41], while Rodríguez-Soto et al. (2019) [27] managed to reduce it to values, ranging between 0.2 and 0.3%.

When storing the previous treatments under controlled humidity and temperature conditions until day 8, a considerable loss of σ_max_ and E was identified in the two TPSs due to their high capacity in water absorption, revealing losses of such tensile properties of 76.7 and 47.0% in TPS1 and 75.5 and 90.6% in TPS2, respectively; however, in the composite materials, lower losses were evident in the σ_max_ of the two treatments (32.6% in BCM1 and 4.4% in BCM2) and E in BCM2, with a loss of 64.1%. The high losses of the tensile properties in the two TPSs are due to their hydrophilic capacity and direct exposure to environment humidity [60], while the lower losses in properties can be related to the possible water vapor barrier that the sheath can generate on the TPS containing the composite material. In the case of the E of BCM1, its value increased by 113.8% due to a greater development of the retrogradation of the matrix it contains, as it has a lower content of fibers and other impurities provided by the starch from the core, allowing the generation of the recrystallization of amylose. It should also be mentioned that when incorporating the starch of the core for the preparation of TPS2 and BCM1, a greater reduction in E was presented when stored for a period of 8 days, relating the greater capacity of water absorption from the presence of pectin, where Holguín-Posso et al. (2024) [24] revelated the same pattern at the same time and storage conditions.

Flexural test. This mechanical test was only applied to composite materials due to their brittle capacity relating to their reduced deformation, finding σ_maxF_ and E_F_ greater than 30 MPa and 3 GPa (Table 2), respectively, which are comparable values to those given by composite materials made of reinforced petroleum-derived plastics, where Adeniyi et al. (2019) [57] reported the flexural properties from different composite materials made of matrices, such as polypropylene, nylon, epoxy resins, and polyester, using plantain pseudostem fibers and considering reinforcement concentrations between 3.7 and 41.0% to give σ_maxF_ values at bending between 8.7 and 50.0 MPa and E at bending between 0.6 and 3.5 GPa, while [61] reported biobased composites from pineapple leaf, olive leaf powder, and an epoxy matrix with σ_maxF_ between 16.4 and 44.4 MPa and E_F_ between 2.0 and 4.8 GPa.

In addition, Adeniyi et al. (2019) [57] indicated that the composites made from plantain fiber, sisal, and epoxy matrix reached σ_maxF_ in intervals between 21.6 and 57.5 MPa. Likewise, the hybrid composites formed from kenaf, pineapple leaf fiber, and high-density polyethylene showed σ_maxF_ between 22 and 28 MPa, with E_F_ that ranges from 1.9 to 2.2 GPa. As the previous results are comparable with those shown in BCM1 and BCM2, the sandwich-like structure is essential for taking advantage of the unidirectional orientation of the lignocellulosic fibers contained in the pseudostem sheaths since Dorigato et al. (2019) [62] reported a biobased composite material from a sandwich structure made from a polyethylene glycol chipboard adhered to a MaterBi^®^ TPS film by compression molding, and a σ_maxF_ of 120.0 MPa and an E_F_ of 14.5 GPa were achieved.

The addition of starch from the pseudostem core contributed to the increase in the reinforcement of BCM2 on day 0 from the presence of fibers and pectin. Likewise, such reinforcement was evidenced by a decrease in deformation, going from 10.4 to 8.3%. As storage time advanced up to day 8, the same pattern as in the tensile properties was evident, generating a reduction in σ_maxF_ values ranging between 67.1 and 73.8%. In E_F_, it was between 78.2 and 80.3%, whereas ε_F_ capacity increased due to higher water absorption (Table 2).

Shore D hardness test. TPS1 had a Shore D hardness of 59.1 (Figure 7), and upon the incorporation of the starch from the core, its value increased to 65.8 Shore D, relating a possible increase in the density of the TPS from the incorporation of residual fibers, with diameters close to 4 µm adhering to the starch from the core [21]. The greater hardness of TPS2 can be related to the possible increase in density given by the presence of lignocellulosic fibers and the secondary interaction between the matrix starch and the pectin present in the core starch [63,64,65]. When comparing other developments in terms of Shore D hardness, Yoksan et al. (2023) [66] reported the hardness of polybutylene adipate–co-terephthalate (PBAT) and PBAT/TPS mixtures from cassava processed through a twin-screw extruder, obtaining values of 45 and between 42 and 52 Shore D, respectively, Tavares et al. (2023) [36] used polybutylene succinate (PBS) and PBS/TPS mixtures through twin-screw extrusion, obtaining values of 64.6 and between 51.3 and 59.3 Shore D, while [67] used mixtures of PBAT and its mixtures with corn TPSs through an internal mixer, reporting values between 37 and 49 Shore D. Adeniyi et al. (2019) [56] reported hardness values between 28.0 and 73.5 Shore D in composite materials consisting of epoxy resin and polypropylene with reinforcements of plantain pseudostem fibers with concentrations between 3.7 and 40.0%.

Comparing the results with the plantain BCM on day 0, some values similar to and higher than those obtained by composite materials made of synthetic biobased polymers with their mixtures with TPSs and synthetic polymers derived from petroleum mixed with fibers from Musaceae plants are obtained. In the case of composite materials, the application of treated sheaths to form the sandwich structure and the incorporation of 1% of short fiber with diameters close to 616.8 µm from the pseudostem contributed to a slight increase in hardness concerning TPS1, which was lower concerning TPS2. The sheaths, by having a porous structure or hollow cavities characteristic of the parenchyma (Figure 3), produce a degree of cushioning at the time of generating the indentation [68].

When the storage time increased to 8 days, a reduction in the hardness values was evident among the different treatments, with a greater decrease in the TPS by having a greater capacity in water absorption than in the composite materials. It is also relevant to mention the lower hardness in the TPS2 and BCM2 treatments when compared to that obtained in TPS1 and BCM1, possibly relating the adherence of pectin to the starch from the core, thus generating its incorporation into the matrix and contributing to the greater capacity in water absorption during storage since pectin is a molecule of greater hygroscopicity concerning starch from the presence of hydroxyl groups and having a lower molecular weight [24,69].

## 4. Conclusions

The use of plantain by-products such as sheaths, sheath fibers, and pseudostem core starch for the development of biobased composite materials contributes to physicochemical, thermal, and mechanical properties. The previously treated sheaths enabled reduced water absorption capacity during 90-day storage under controlled conditions and increased the thermal stability of the high glycerin content phases, the σ_max_, and the tensile and flexural E at day 0 when compared to the respective TPS.

With regard to core starch, BCM2 kept a higher E of tension and flexural properties, which is related to the possible reinforcing action coming from the fibers and pectin residuality, although on day 8 of storage, the Shore D hardness, the σ_max_, and the E of tension and flexural strength had a greater reduction in their values due to the greater capacity for water absorption from the easy moistening of the pectin that it may contain. It should be highlighted that the values of tensile, flexural, and hardness properties of the freshly processed BCM1 and BCM2 are comparable with those found in some composite materials based on synthetic polymeric matrices and reinforced with lignocellulosic fibers when obtained freshly processed and that one alternative to extend their outstanding properties during storage is the use of a coating constituted by an apolar resin that provides a humidity barrier.

The industrial scalability of the transformation process proposed in this research is limited by the current polymeric material processing machinery available on the global market. To achieve a continuous, low-cost process, there is a need for the design, construction, and commissioning of specialized equipment based on hot molding compression techniques.

## Figures and Tables

**Table 1:** TPSs and biobased composite material treatments involved in the study.

Treatment	Glycerol (g/100 g)	Pulp and Peel Starch (g/100 g)	Core Starch (g/100 g)	Sheath Short Fiber (g/100 g)	Reinforcement with Treated Sheath (g/100 g)
TPS1	35.0	65.0	0	0	0
TPS2	35.0	60.0	5.0	0	0
BCM1	22.1	40.3	0	0.6	37
BCM2	22.1	37.1	3.2	0.6	37

**Table 2:** Flexural properties of TPSs and BCMs.

Sample	Day	σ_maxF_ (MPa)	E_F_ (GPa)	ε _F_ (%)
BCM1	0	32.4 ± 7.6	3.3 ± 0.9	10.4 ± 5.6
	8	10.7 ± 3.3	0.7 ± 0.2	16.5 ± 6.0
BCM2	0	53.8 ± 8.7	3.5 ± 1.6	8.3 ± 5.6
	8	14.1 ± 5.1	0.7 ± 0.3	15.2 ± 1.4

## Data Availability

Data are contained within the article.
